# An Inductive Sensor for Two-Dimensional Displacement Measurement

**DOI:** 10.3390/s20071819

**Published:** 2020-03-25

**Authors:** Liang Wu, Shi Xu, Ziqiang Zhong, Chuan Mou, Xinda Wang

**Affiliations:** Engineering Research Center of Mechanical Testing Technology and Equipment (Ministry of Education), Chongqing University of Technology, Chongqing 401135, China; xushi@cqut.edu.cn (S.X.); zzq@2018.cqut.edu.cn (Z.Z.); mouchuan@2018.cqut.edu.cn (C.M.); wangxinda@2019.cqut.edu.cn (X.W.)

**Keywords:** two-dimensional displacement measurement, inductive sensor, spiral coils, pulsating magnetic field, CORDIC algorithm

## Abstract

The simultaneous and independent measurements of two-dimensional (2D) displacements are significant for 2D positioning. Here a planar inductive sensor which is based on the principle of electromagnetic induction is proposed. The sensor is composed of a primary coil and a secondary coil. The primary coil consists of an array of planar spiral coils which are arranged as an m × n matrix. The primary coil is supplied with 4 kHz alternating current to generate an array of pulsating magnetic field. The secondary coil contains four spiral coils which are arranged as a 2 × 2 matrix. Thereby, four roads of modulated signals whose amplitudes vary with displacements of the secondary coil along x- and y-axis are induced. An algorithm based on the Coordinate Rotation Digital Computer algorithm is introduced to resolve the planar displacements. The structure and working principle of the sensor are proposed firstly. Then, the finite element analysis of the electromagnetic model and the numerical simulation of the algorithm are given. An experiment has been performed on a sensor prototype and the results show that the proposed scheme is feasible. Measurement error analysis of the sensor has been pursued at the end of the paper.

## 1. Introduction

The development of semiconductors and micro-electronics industry has been attracting great attention in the area of rapid and precise two-dimensional (2D) positioning, where the motion control and the 2D displacement measurement are vital [1,2]. Generally, the high-resolution actuators and high precision instruments are the critical components. As feedback, the instruments with high resolution and precision are especially necessary [3,4].

A pair of laser interferometers or linear encoders which are mounted orthogonally are usually utilized for the 2D displacement measurement. Michelson laser interferometer is mostly used, and it can realize a long-range measurement with nanometer resolution. SIOS technic GmbH Company developed an interferometer which had the resolution of 5 pm within 80 m range [5]. However, a second interferometer is necessary for 2D displacement measurement which makes the measuring system bigger and more costly. Moreover, the measurement result may also be influenced by the beam interference, humidity and temperature fluctuation. On the other hand, two linear encoders are usually mounted orthogonally. Although the best commercial optical linear encoder can realize the accuracy of ±140 nm within 280 mm range [6], the optical encoder is sensitive to the contamination and strong vibration [7]. In addition, the Abbe error is easy to be introduced by the unbalanced installation and perpendicularity of motion axes. 

The optical planar encoders have been developed for the 2D displacement measurement. The study shown in [8] described a kind of planar laser diffraction encoder with Littrow configuration. Using the Doppler Effect, 2D displacements were obtained. The measurement accuracies of both directions were 20 nm within the range of 25 mm. The authors of [9] proposed a measurement system with three vertical cavity surface emitting lasers and three photodiodes. The resolutions of *x* and *y* were 20 nm and 40 nm within 0.4 mm and 1.8 mm range. A novel system that realized a single-spot 2D displacement measurement of a non-cooperative target was reported in [10]. The measurement range was 500 μm and the accuracy was submicron. In [11], the authors developed a fiber optic sensor, and the linear sensitivity was equal to 11.20 ± 0.12 mV/μm within a range of 13.03 mm. A 2D planar grating encoder based on the double diffraction grating principle was reported in [12]. The encoder included a combined transmission rectangular grating and 2D reflective type rectangular grating. The validity of the encoder was verified by experiments. Furthermore, a three axis grating encoder was introduced in [13]. The encoder was composed of a planar scale grating and an optical reading head. A planar reference grating was integrated in the optical reading head. The x-axial measurement error was 4.16 μm in the range of 495.8 μm, while the z-axial measurement error was 0.41 μm in the range of 6.868 μm [14]. Though the surface encoder is easy to embed in the motion stage, the optical modules are complicated and hard to align rigidly. Moreover, the distance between the optical head and scale grating is so tight that the quality of optical signals is easily influenced by the mechanical wobble and vibration. Hence, the optoelectronic signals may decay or disappear. On the other hand, the scale grating over a large area is difficult to manufacture with high accuracy [15].

2D capacitive displacement sensors have also been developed. A planar capacitive sensor for 2D long-range displacement measurement was proposed in [16]. The sensor consisted of a moving plate and a fixed plate, which were composed of arrays of capacitor plates. The resolution was 0.308 μm. The equivalent displacement error caused by fringe effects was 237 μm, and the equivalent displacement interpretation error was 141 μm. Moreover, a novel phase-shift arctangent interpolation method was utilized to decrease waveform errors from 4% to 1.72% [17]. A planar position sensor based on mono sensing electrode and hybrid-frequency excitation was proposed in [18]. The displacement sensitivity on an order was 1.5 mV per micron and measurement repeatability was better than 0.002 mm during the range of 256 mm^2^. However, the permittivity between two electrode plates is sensitive to the humidity, temperature, and air pressure. Therefore, the capacitive sensor is rarely employed in the harsh environment.

There are some researches on the 2D magnetic and inductive sensors. Three degrees of freedom displacement measurement system composed of Hall sensor was proposed in [19]. The measurement error of x-axis was 4.6 μm, and that of y-axis was 4.8 μm after compensation. The measurement range was only 4 mm. [20] presented GaussSense, which was a novel back-of-device sensing technique. A 2 mm-thick Hall sensor grid was developed to sense magnets that were embedded in the stylus. The GaussSense board was developed to identify not only the position, but also the tilt angle and applied pressure of the stylus. An inductive sensor with planar coils was proposed in [21]. The magnetic field was generated by meander coils, while four flat coils output signals which were related with *x* and *y*. The resolution of the sensor was 10 μm, but there were no further researches. A planar displacement sensor with inductive spiral coils was presented in [22]. The experimental results showed that the prototype with spiral coils had superior characteristics than that of meander type in some respects such as useful signal and useful measurement range. A 2D sensor with spiral coils was developed in [23], and the measurement range was 140 mm × 140 mm while the linearity was 1% for one pitch. Two magnetic fields which travel orthogonally were generated by four groups of spiral coils. The phase comparison method was utilized to obtain the displacements. The authors of [24] proposed an inductive displacement sensor for small displacements (less than 0.5 mm) in the plane. The sensor consisted of two sensor elements, each having a pair of meander coils. The optimal useful measurement range was obtained if the gap of 0.23 mm was inserted in the stationary coil [25]. In another method, spiral coils have been utilized for displacement sensing. A planar linear variable differential transformer with spiral coils was reported in [26]. The measurement range was 70 mm and R.M.S. error was 0.8%. A planar spiral coil-based inductive displacement sensor was presented in [27]. The sensor was composed of a fixed planar coil and a movable U-shaped magnetic core. The shape of inductance versus displacement *x* was sinusoidal accordingly. The worst case error was 0.2% and the resolution was 6.5 μm. In this paper, a novel and easily manufactured 2D inductive sensor using spiral coils is proposed. The spiral coils are arranged in plane to make the effective magnetic field strength vary as a planar standing wave. This approach not only brings in high sensitivity but also reduce the harmonic components of the measurement results. The paper is organized as follows: in Section 2, the basic structure and working principle of the sensor are described. Then, finite element analysis of the sensor model and numerical simulation of the algorithm are presented in Section 3. Section 4 shows the experiment with a sensor prototype and experimental results are discussed. Finally, conclusions are summarized in Section 5.

## 2. Structure and Measurement Principles

### 2.1. Structure of the Sensor

**Figure 1:**
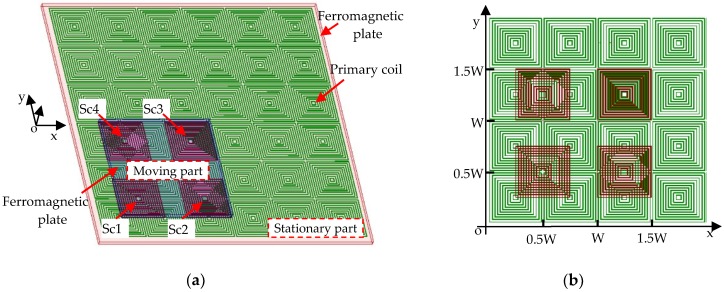
(**a**) Structure diagram of the 2Dsensor. (**b**) Arrangement of primary and secondary coil.

As shown in Figure 1a, the sensor consists of a primary coil and a secondary coil. The primary coil is a planar array of spiral coils, which are arranged as an m × n matrix. The winding directions of adjacent spiral coils are opposite, and all of them are connected in series. Given that the layout period of the primary coil is considered to be a pitch (W), the center to center distance between two successive spiral coils along x- or y-axis is half pitch. The primary coil is supplied with the alternating current, and an array of pulsating magnetic field above the primary coil is generated. The secondary coil has four independent and identical spiral coils which are named as Sc1, Sc2, Sc3 and Sc4. The length of the outmost turn of each spiral coil is half pitch along the x- and y-axis. The arrangement of the primary and secondary coil is shown in Figure 1b.

In order to describe the relationship between the primary and secondary coil clearly, the spiral coils are simplified into multi-turn square coils. Sc1 and Sc2 are aligned along the x-axis, and the center distance between them is 0.75 W. Meanwhile, Sc1 and Sc4 are aligned along y-axis, and the center distance between them is 0.75 W too. Sc3 is aligned with Sc4 along x-axis and aligned with Sc2 along y-axis. The air gap between the primary coil and secondary coil is kept small to make sure that they can move with respect to each other. In order to enhance and restrain the magnetic field, a ferromagnetic plate is stuck on the back of the primary and secondary coil respectively. The plate with the primary coil constitutes the stationary part, while the plate with secondary coil constitutes the moving part.

### 2.2. Electro-Magnetic Induction

**Figure 2:**
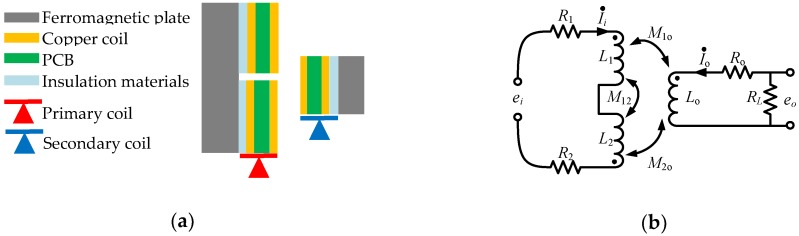
(**a**) Cross section scheme of the sensing element. (**b**) The equivalent circuit of the sensing element.

The basic unit of the primary and secondary coil is a spiral coil, and the mutual inductance between the spiral coils plays a significant role during electro-magnetic induction. Taking two adjacent spiral coils in x- or y-axis as a sensing element, the cross section scheme and equivalent circuit model are shown in Figure 2.

Each spiral coil is equivalent to a resistance and an inductance. According to the Kirchhoff’s law, the equivalent circuits of spiral coils in primary and secondary coil can be expressed as below.

(1) $$\{\begin{array}{l} \dot{I_{0}}\left[R_{1}+R_{2}+j\omega \left(L_{1}+L_{2}-{2M}_{12}\right)\right]+j\omega \dot{I_{0}}{(M}_{1o}{-M}_{2o}{)=e}_{i}\\ \dot{I_{0}}\left(R_{o}{+R}_{L}{+j\omega L}_{o}\right)+j\omega \dot{I_{i}}{(M}_{1o}{-M}_{2o})=0\\ \dot{I_{0}}R_{L}=e_{o} \end{array}$$

where *e_i_* is the input electromotive force (EMF) of the primary coil, *ω* is the frequency of the input EMF, $\dot{I}$*_i_* and $\dot{I}$*_o_* are the phasor form of driving current and output current respectively, *R*_1_, *R*_2_ and *L*_1_, *L*_2_ are the resistances and self-inductance of two adjacent spiral coils, *R*_o_ and *L*_o_ are the resistance and self-inductance of spiral coil in the secondary coil, *M*_12_ is the mutual inductance between two adjacent spiral coils, *M*_1o_ and *M*_2o_ are the mutual inductances between two adjacent spiral coils and secondary coil, *R*_L_ is the load resistance. 

According to Equation (1), the output EMF is shown as below.

(2) $$e_{o}{=j\omega (M}_{2o}{-M}_{1o}{)R}_{L}e_{i}{\left\{\begin{array}{c} \left(R_{o}{+R}_{L}{+j\omega L}_{o}\right)\left[R_{1}{+R}_{2}+j\omega \left(L_{1}{+L}_{2}{-2M}_{12}\right)\right] \end{array}-{\left(j\omega \right)}^{2}{{(M}_{1o}{-M}_{2o})}^{2}\right\}}^{-1}$$

For the primary and secondary coil, the resistance, self-inductance and the mutual inductances of spiral coils in primary coils are fixed with the structural parameters. However, the mutual inductance between primary and secondary coil is determined by coupling area and vertical distance [28]. When the secondary coil moves in plane, the output EMF varies with planar displacement of the secondary coil. 

In order to describe the process of eletro-magenetic induction in details, the magnetic field analysis is introduced. A spiral coil is simplified into a multi-turn square coil. According to the Biot-Savart Law, the magnetic field above the planar coil is contributed by the current flow in wires. Because the secondary coil is paralleled with the primary coil, the effective magnetic flux density is the vector component along z-axis [29]. Taking the center of multi-turn square coil as the origin of the Cartesian coordinate system, the effective magnetic density distributed by multi-turn square coil at point *P*(*x*,*y*,*z*_0_) is shown as Equation (3).

(3) $$B(x,y,z_{0},t)=\frac{\mu_{0}\sin\omega t}{4\pi }\Sigma_{j=1}^{o}\{\begin{array}{c} \frac{a_{j}-x}{{\left(a_{j}-x\right)}^{2}+{z_{0}}^{2}}[\frac{a_{j}-y}{\sqrt{{\left(a_{j}-x\right)}^{2}+{\left(a_{j}-y\right)}^{2}+{z_{0}}^{2}}}+\frac{a_{j}+y}{\sqrt{{\left(a_{j}-x\right)}^{2}+{\left(a_{j}+y\right)}^{2}+{z_{0}}^{2}}}]+\\ \frac{a_{j}-y}{{\left(a_{j}-y\right)}^{2}+{z_{0}}^{2}}[\frac{a_{j}+x}{\sqrt{{\left(a_{j}+x\right)}^{2}+{\left(a_{j}-y\right)}^{2}+{z_{0}}^{2}}}+\frac{a_{j}-x}{\sqrt{{\left(a_{j}-x\right)}^{2}+{\left(a_{j}-y\right)}^{2}+{z_{0}}^{2}}}]+\\ \frac{a_{j}+x}{{\left(a_{j}+x\right)}^{2}+{z_{0}}^{2}}[\frac{a_{j}+y}{\sqrt{{\left(a_{j}+x\right)}^{2}+{\left(a_{j}+y\right)}^{2}+{z_{0}}^{2}}}+\frac{a_{j}-y}{\sqrt{{\left(a_{j}+x\right)}^{2}+{\left(a_{j}-y\right)}^{2}+{z_{0}}^{2}}}]+\\ \frac{a_{j}+y}{{\left(a_{j}+y\right)}^{2}+{z_{0}}^{2}}[\frac{a_{j}-y}{\sqrt{{\left(a_{j}-x\right)}^{2}+{\left(a_{j}+y\right)}^{2}+{z_{0}}^{2}}}+\frac{a_{j}+x}{\sqrt{{\left(a_{j}+x\right)}^{2}+{\left(a_{j}+y\right)}^{2}+{z_{0}}^{2}}}]+ \end{array}\}$$

where *μ*_0_ is the air permeability, *ω* is the angular frequency of alternating current, *z*_0_ is the fixed vertical distance above the square coil, *a_j_* is the length of *j*^th^ turn of the square coil, *o* is the turns of the square coil.

*B*(*x*,*y*,*z*_0_,*t*) can be decomposed into many components due to the central symmetry. The main component of the magnetic flux density is expressed as below.

(4) $$B_{1}x,y,t=k\sin\omega t\sin(\frac{2\pi }{W}{x+\psi }_{A})\sin(\frac{2\pi }{W}{y+\psi }_{B})$$

where *k* is the amplitude of the main component, *W* is the pitch, *ψ_A_* is the initial phase along x-axis, *ψ_B_* is the initial phase along y-axis.

**Figure 3:**
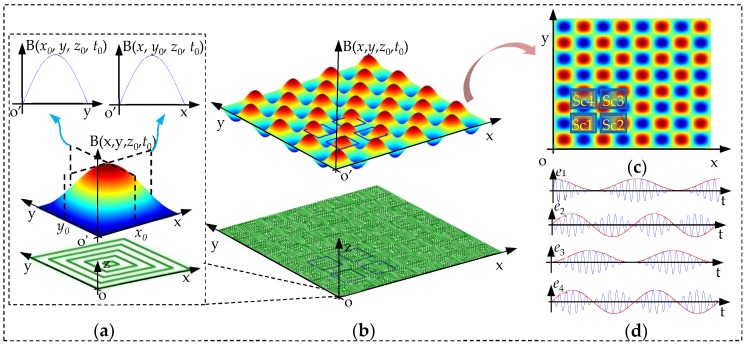
Generation of the electrical signals. (**a**) Spiral coil and transient magnetic field at *z*_0_. (**b**) Primary coil and an array of pulsating magnetic field at *z*_0_. (**c**) Secondary coil and the top view of the magnetic field. (**d**) Induced electromotive forces (EMFs) of secondary coil.

As shown in Figure 3a, the main component of the magnetic flux density along y-axis at *x* = *x*_0_ is similar to a sinusoidal curve, while the magnetic flux density along x-axis at *y* = *y*_0_ is similar to a sinusoidal curve too. Besides, the amplitudes of the sinusoidal curves are determined by *y* and *x* respectively. Thereby, the magnetic flux density generated by the primary coil is similar to an array of pulsating magnetic field as shown as Figure 3b. Secondary coil consists of four spiral coils, and the magnetic flux for each one is the integral form of magnetic density at different positions. Taking the outmost turn of the secondary coil as an example, the interval is [*x_p_*, *x_p_* + 0.5W] along x-axis, and [*y_p_*, *y_p_* + 0.5W] along y-axis. The process of integration is expressed as Equation (5). As shown as Figure 3c, the initial positions of four spiral coils are different. (*x_p_*, *y_p_*) for Sc1~Sc4 are (*x*, *y*), (*x* + 0.75W, *y*), (*x* + 0.75W, *y* + 0.75W), (*x*, *y* + 0.75W). Therefore, the magnetic flux of Sc1~Sc4 are expressed as Equation (6).

(5) $$\varphi =\int_{y_{p}}^{y_{p}+0.5W}\int_{x_{p}}^{x_{p}+0.5W}k\sin\omega t\sin(\frac{2\pi }{W}{\delta +\psi }_{A})\sin(\frac{2\pi }{W}{\varepsilon +\psi }_{B})d\delta d\varepsilon$$

where *p* counts from 1 to 4, *δ* is the variable of integration along x-axis, *ε* is the variable of integration along the y-axis.

(6) $$\left\{ \begin{array}{l} \varphi_{1}=\frac{kW^{2}}{\pi^{2}}\sin\omega t\cos(\frac{2\pi }{W}x+\psi_{A})\cos(\frac{2\pi }{W}y+\psi_{B})\\ \varphi_{2}=\frac{kW^{2}}{\pi^{2}}\sin\omega t\sin(\frac{2\pi }{W}x+\psi_{A})\cos(\frac{2\pi }{W}y+\psi_{B})\\ \varphi_{3}=\frac{kW^{2}}{\pi^{2}}\sin\omega t\sin(\frac{2\pi }{W}x+\psi_{A})\sin(\frac{2\pi }{W}y+\psi_{B})\\ \varphi_{4}=\frac{kW^{2}}{\pi^{2}}\sin\omega t\cos(\frac{2\pi }{W}x+\psi_{A})\sin(\frac{2\pi }{W}y+\psi_{B}) \end{array}\right.$$

The induced EMFs of Sc1~Sc4 are the differential form of the magnetic flux, which are shown as below.

(7) $$\left\{ \begin{array}{l} e_{1}=k_{e}\cos\omega t\cos(\frac{2\pi }{W}x+\psi_{A})\cos(\frac{2\pi }{W}y+\psi_{B})\\ e_{2}=k_{e}\cos\omega t\sin(\frac{2\pi }{W}x+\psi_{A})\cos(\frac{2\pi }{W}y+\psi_{B})\\ e_{3}=k_{e}\cos\omega t\sin(\frac{2\pi }{W}x+\psi_{A})\sin(\frac{2\pi }{W}y+\psi_{B})\\ e_{4}=k_{e}\cos\omega t\cos(\frac{2\pi }{W}x+\psi_{A})\sin(\frac{2\pi }{W}y+\psi_{B}) \end{array}\right.$$

where *k_e_* = −*kW*^2^*ωπ*^−2^. The carrier signal of EMFs is cos*ωt*, and the modulation signals are related with *x* and *y*. When the secondary coil moves along *y* = *x* (i.e., diagonally), the comparison of four EMFs is shown in Figure 3d. The abscissa indexes time and the ordinate indexes the amplitude. It can be learned that the amplitudes of carrier signals vary with the displacements of *x* and *y*.

### 2.3. Resolving of Displacements

**Figure 4:**
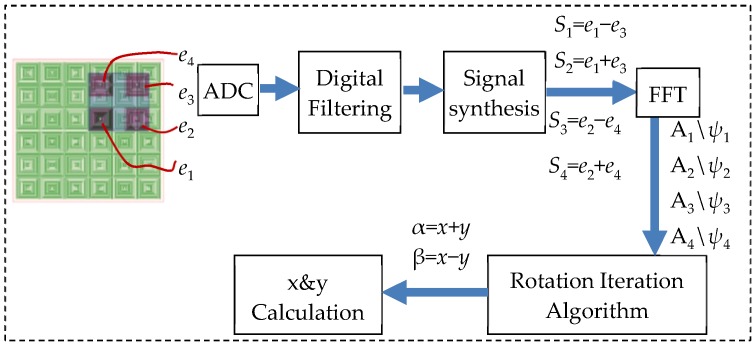
Processing of the electrical signals.

The processing of output signals is shown in Figure 4. Firstly, four channels of analog-digital converter (ADC) are utilized to sample the signals. Then, the digital filtering is employed to reduce the noise jamming. In the module of signal synthesis, *e*_1_~*e*_4_ are computed as Equation (8) and *S*_1_~*S*_4_ are outputted.

(8) $$\left\{ \begin{array}{l} S_{1}=e_{1}-e_{3}=k_{e}cos\omega t\cos[\frac{2\pi }{W}(x+y)+\psi_{A}+\psi_{B}]\\ S_{2}=e_{1}+e_{3}=k_{e}cos\omega t\cos[\frac{2\pi }{W}(x-y)+\psi_{A}-\psi_{B}]\\ S_{3}=e_{2}-e_{4}=k_{e}cos\omega t\sin[\frac{2\pi }{W}(x-y)+\psi_{A}-\psi_{B}]\\ S_{4}=e_{2}+e_{4}=k_{e}cos\omega t\sin[\frac{2\pi }{W}(x+y)+\psi_{A}+\psi_{B}] \end{array}\right.$$

In addition, four roads of signals are processed by the fast Fourier transform (FFT) to obtain the amplitudes and initial phases. The amplitudes are expressed as Equation (9), where *α* is *x* + *y* and *β* is *x* − *y*.

(9) $$\{\begin{array}{l} A_{1}=\left|k_{e}\cos(\frac{2\pi }{W}{\alpha +\psi }_{A}{+\psi }_{B})\right|\\ A_{2}=\left|k_{e}\cos(\frac{2\pi }{W}{\beta +\psi }_{A}{-\psi }_{B})\right|\\ A_{3}=\left|k_{e}sin(\frac{2\pi }{W}{\beta +\psi }_{A}{-\psi }_{B})\right|\\ A_{4}=\left|k_{e}\sin(\frac{2\pi }{W}{\alpha +\psi }_{A}{+\psi }_{B})\right| \end{array}$$

In order to obtain *α* and *β*, the coordinate rotation digital computer (CORDIC) algorithm is utilized [30]. For the vector (*a*_0_, *b*_0_), the phase angle *θ* can be computed by aligning the vector along a- coordinate. The micro-rotation *ζ_i_* which is equal to arctan2*^−i^* drives the b-coordinate to zero. The control variable *d_i_* which is the reverse of the sign of *a_i_b_i_* determines the direction of micro-rotations. If *d_i_* is positive, the direction of micro-rotation is clockwise, while it is anticlockwise if *d_i_* is negative. After the *q*^th^ rotation, the vector is shown as Equation (10). Meanwhile, the residual angle shown as Equation (11) approaches the resolved phase.

(10) $$\left[\begin{array}{c} a_{q+1}\\ b_{q+1} \end{array}]=\prod_{i=0}^{q}{\cos\xi }_{i}\prod_{i=0}^{q}[\begin{array}{cc} 1 & {-d}_{i}{\tan\xi }_{i}\\ d_{i}{\tan\xi }_{i} & 1 \end{array}][\begin{array}{c} a_{i}\\ b_{i} \end{array}]=\prod_{i=0}^{q}\frac{1}{\sqrt{{1+2}^{-2i}}}\prod_{i=0}^{q}[\begin{array}{cc} 1 & {-d}_{i}2^{-i}\\ d_{i}2^{-i} & 1 \end{array}][\begin{array}{c} a_{i}\\ b_{i} \end{array}\right]$$

(11) $$\theta_{i+1}=\theta_{i}-d_{i}\xi_{i}$$

Taking *A*_1_ and *A*_4_ as *a*_0_ and *b*_0_, α can be acquired by the proposed algorithm. Meanwhile, *a*_0_ and *b*_0_ are assigned with *A*_2_ and *A*_3_, and then *β* is acquired. As shown as Equation (12), the displacements *x* and *y* are calculated by solving the system of linear equations. The detailed description of the resolving process is described in Section 3.2.

(12) $$\{\begin{array}{l} x=\frac{W}{2\pi }\alpha -\frac{W}{2\pi }\psi_{A}\\ y=\frac{W}{2\pi }\beta -\frac{W}{2\pi }\psi_{B} \end{array}$$

## 3. Simulation of Sensor Model

### 3.1. FEA Simulation

A 3D model of the sensor is designed and simulated in Finite Element Analysis (FEA) software ANSYS Maxwell 15.0. The electrical and mechanical parameters of the model are described in Table 1. In order to simulate the real process of electro-magnetic induction, some parameters are set ideally. For example, the resistance of Sc1~Sc4 is set to be 1 GΩ which make sure the current of the secondary coil is tiny. Therefore, the induced EMFs are mostly acquired. In addition, the thickness of air-gap between the primary coil and ferromagnetic plate is set at 0.1 mm, and so is the air-gap between the secondary coil and ferromagnetic plate. The air-gap is kept for the insulating material between the conductor and ferromagnetic material.

**Figure 5:**
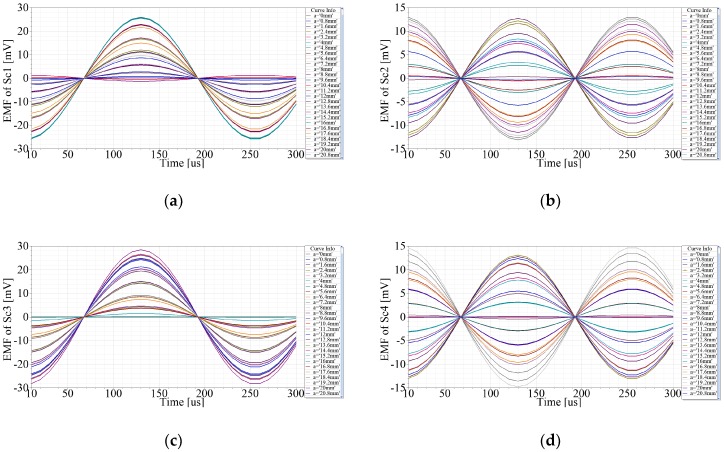
Finite Element Analysis (FEA) simulation results, EMFs of Sc1~Sc4 when primary coil moves 20.8 mm with a step of 0.8 mm. (**a**) EMF of Sc1. (**b**) EMF of Sc2. (**c**) EMF of Sc3. (**d**) EMF of Sc4.

**Figure 6:**
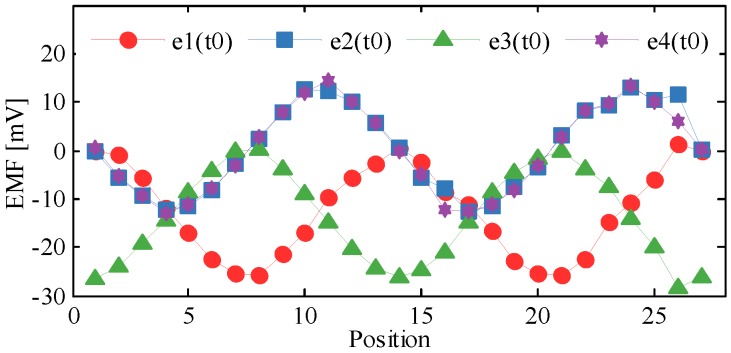
Comparison of EMFs vary with displacements at *t*_0_ = 250 μs.

The moving path of the secondary coil is *y* = *x*. The displacements in x- and y-aixs are both 20.8 mm with a step of 0.8 mm respectively, so there are 27 positions at all. The simulation results are shown in Figure 5a–d. The abscissa indicates time, while the ordinate indicates the induced EMFs of Sc1~Sc4. Each curve describes that the EMF varies with time at a fixed position, so the amplitudes of the EMFs are related with displacements. The comparison of EMFs which vary with displacements at *t*_0_ = 250 μs is shown in Figure 6. By adjusting the initial position, the initial phases of *e*_1_~*e*_4_ can be zero. Hence, the EMFs of Sc1~Sc4 at *t* = *t*_0_ are expressed as Equation (13), where *γ* is the linear step of *x* or *y*. The amplitudes of *e*_1_(*t*_0_)~ *e*_4_(*t*_0_) vary twice during the simulation, which are similar to the curves of Figure 6. According to (13), *e*_1_(*t*_0_) and *e*_3_(*t*_0_) have direct components which keep the curves mostly below zero, and the phase difference between them is π, while *e*_2_(*t*_0_) is almost the same as *e*_4_(*t*_0_). Because of the simulation deviations, the curves of *e*_1_(*t*_0_)~ *e*_4_(*t*_0_) have some distortions.

(13) $$\{\begin{array}{l} e_{1}{(t}_{0})=\frac{k_{e}}{2}{\cos\omega t}_{0}[1+\cos(\frac{2\pi }{W}2\gamma )]\\ e_{2}{(t}_{0})=\frac{k_{e}}{2}{\cos\omega t}_{0}\sin(\frac{2\pi }{W}2\gamma )\\ e_{3}{(t}_{0})=\frac{k_{e}}{2}{\cos\omega t}_{0}[1-\cos(\frac{2\pi }{W}2\gamma )]\\ e_{4}{(t}_{0})=\frac{k_{e}}{2}{\cos\omega t}_{0}\sin(\frac{2\pi }{W}2\gamma ) \end{array}$$

### 3.2. Numerical Simulation

**Figure 7:**
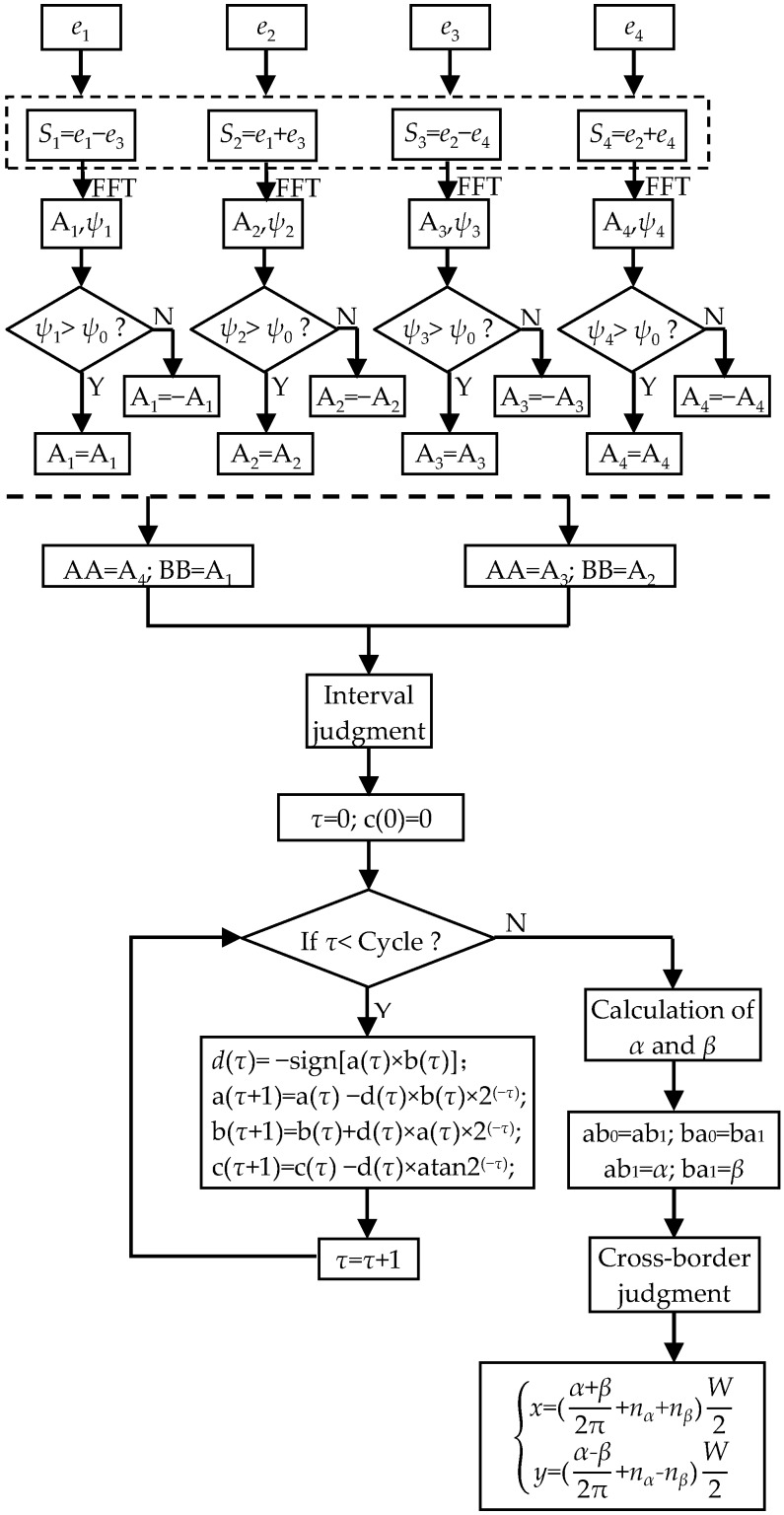
Algorithm flowchart.

Taking the simulation results of FEA as the input, the flowchart of the resolving process of *x* and *y* is shown in Figure 7. 

**Figure 8:**
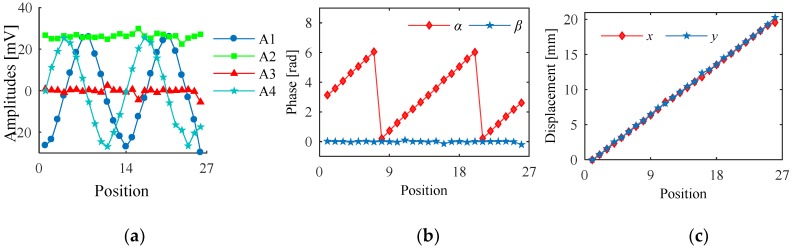
Calculation results based on the algorithm. (**a**) Amplitudes of *S*_1_~*S*_4_ that vary with displacements. (**b**) Variations of *α* and *β*. (**c**) Calculation results of *x* and *y*.

Firstly, the outputs of Sc1~Sc4 are processed by Equation (8), and *S*_1_~*S*_4_ are obtained. Secondly, the FFT algorithm is utilized to acquire the amplitudes and phases of the signals. In the third step, *ψ*_0_ is introduced to be the reference phase which determines the signs of *A*_1_~*A*_4_ by phase comparisons. *A*_1_~*A_4_* of *S*_1_~*S*_4_ are calculated from FEA results, as shown in Figure 8a. Because the moving path of the secondary coil is *y* = *x*, the variables of *A*_1_ and *A*_4_ are equal to 2*x*, and the variables of *A*_2_ and *A*_3_ are 0. Hence, the curves of *A*_1_ and *A*_4_ vary twice in one pitch. Meanwhile, *A*_2_ is almost the same as the maximum value, and *A*_3_ is close to zero. Fourthly, variables AA and BB are assigned with A_4_ and A_1_, and the initial values a(0) and b(0) are determined according to different conditions shown in Table 2. Then, the CORDIC algorithm is utilized. Cycle is the number of iterations which is 50, and *d*(*τ*) determines the direction of rotation. After the iterations, c(50) is the evaluated value. The relationship between *α* and c(50) is shown in Table 3. Meanwhile, A_2_ and A_3_ are processed in the same way, and *β* is obtained. The calculation of *α* and *β* at different positions are shown in Figure 8b. Fifthly, a cross-border judgment is utilized to extend the range of *α* and *β*. ab_1_ and ba_1_ are the current values of *α* and *β*, while ab_0_ and ba_0_ are the last values of *α* and *β*. As shown as Table 3, the variation range of *α* and *β* can be separated into eight cases, and the number of periods (*n_α_* and *n_β_*) increases and decreases accordingly. Sixthly, *x* and *y* are obtained by solving the systems of linear equations. The calculation results of *x* and *y* are shown in Figure 8c.

## 4. Experiment and Results

**Figure 9:**
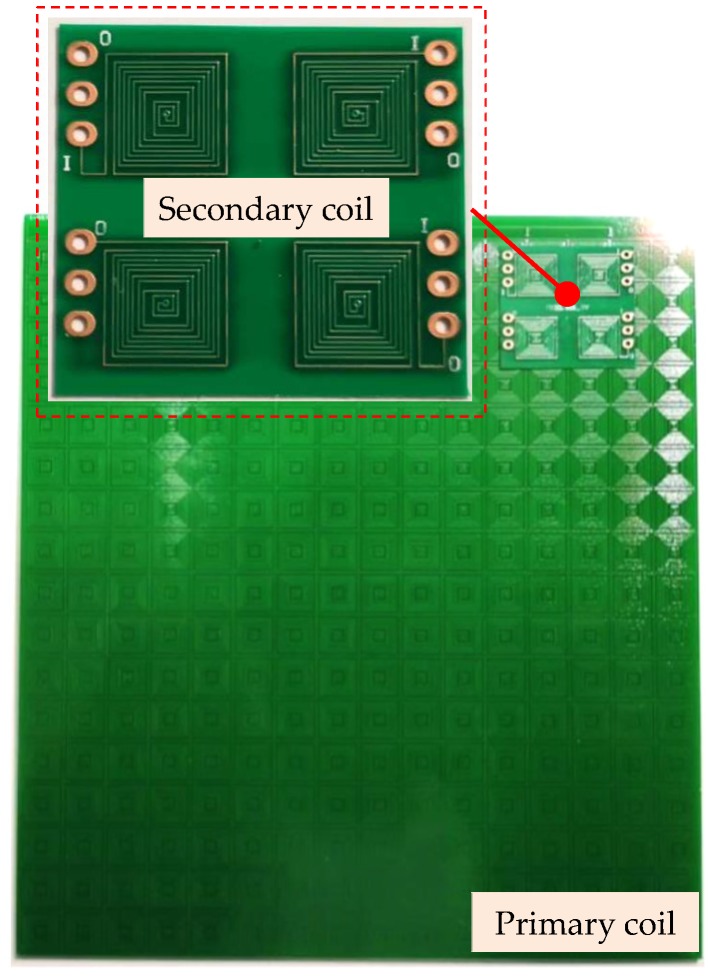
The Sensor prototype.

As shown in Figure 9, a sensor prototype is developed and fabricated by Printed Circuit Board (PCB) technology. The size of the primary coil is 168 mm × 185 mm × 1.6 mm, and the size of the secondary coil is 35 mm × 35 mm × 1.6 mm. Therefore, the measurement range of the sensor is 133 mm × 150 mm. Both the primary and secondary coil are 2-layer PCB. There are holes at the center of each spiral coil to connect the spiral coils of the top layer and bottom layer. The numbers of spiral coils of the primary coil are 16 × 17. The resistance of the primary coil is 7.66 Ω, and the self-inductance is 7.79 μH. Meanwhile, the resistance of the secondary coil is 2.6 Ω, and the self-inductance is 0.44 μH. The other parameters of the primary and secondary coil are the same as the simulation model shown in Table 1.

**Figure 10:**
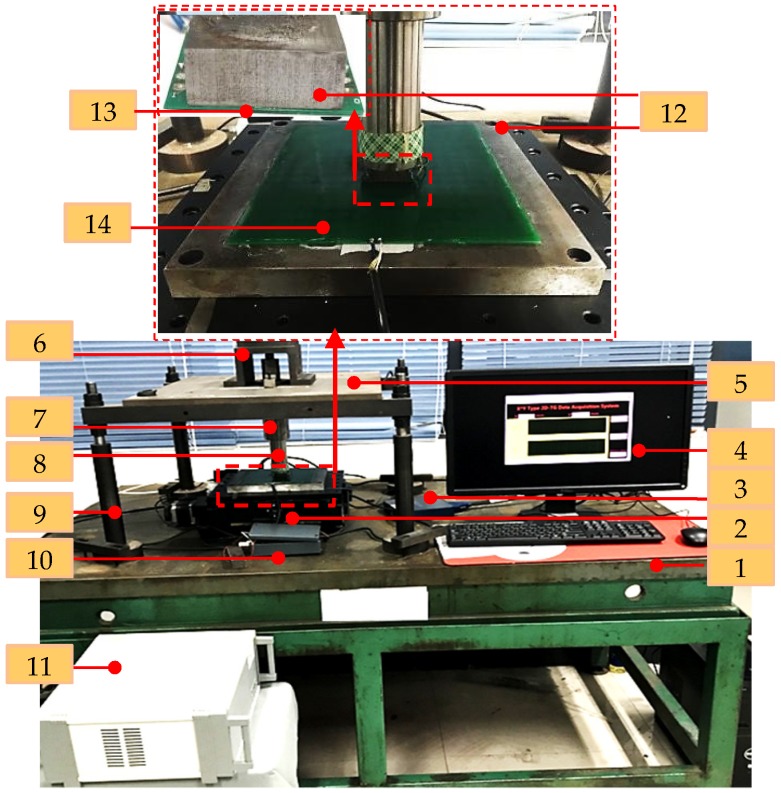
Experimental setup. **1**.base plate; **2**.2D precision positioning stage; **3**.NI data acquisition system (DAQ); **4**.display interface; **5**.upper baseplate; **6**.upper stem; **7**.linear bearing; **8**.feed rod; **9**.steelstems; **10**.exciting circuit; **11**.control box of the stage; **12**.ferromagnetic plate; **13**.secondary coil; 14.primary coil.

The experimental platform shown in Figure 10 consists of a sensor prototype, a 2D precision positioning stage, a data acquisition system (DAQ), etc. The material of the ferromagnetic plate is steel_1045, and the flatness of the plate is kept below 1 μm by abrasive machining. The size of the plate used for primary coil is 220 mm × 220 mm × 15 mm, and it is mounted on the surface of the 2D precision positioning stage. Meanwhile, the size of the plate used for secondary coil is 22 mm × 22 mm × 15 mm, and it is mounted on the end of the feed rod. The stage which is driven by two servo motors can move along the x- and y-axis simultaneously. A pair of Renishaw grating scales coupled with reading heads are mounted on the orthogonal edges of the stage. The measurement values of gratings are not only used for the position feedback, but also used for the calibration of the sensor prototype. A linear bearing is utilized to make sure the feed rod can move along z-axis, so the distance between primary and secondary coil can be adjusted. The linear bearing is mounted on the upper baseplate which is installed on the base plate with four steelstems.

An exciting circuit is developed to drive the primary coil. The alternating current signal is generated by the DAC module of MCU (Model: STM32F407ZGT6). Then, the direct current bias, band-pass filtering module and power amplification circuits are utilized to drive the primary coil. The amplitude of the driving signal is 3.2 V. The outputs of secondary coil are obtained by NI DAQ (Model: PCIe-6259). The sampling frequency for each channel is set to be 240 kHz, and the resolution of the DAQ is 16 bits. Therefore, the resolution of the sensor is about 0.3 μm. The outputs of secondary coil are processed in NI DAQ and the resolving process is realized by Labview 2011.

**Figure 11:**
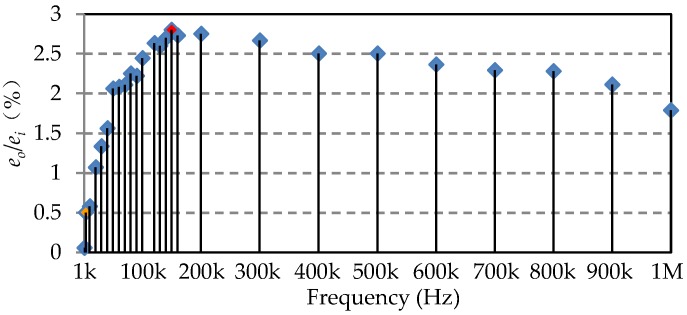
Frequency response of the sensor prototype. The plot is obtained by sweeping the frequency of the driving EMF (*e_i_*) to the primary coil and recording the induced EMF (*e_o_*) in the secondary coil.

In order to choose the driving frequency, a frequency sweep test is operated by varying the frequency of the driving voltage of primary coil and measuring the induced voltage of secondary coil. The results obtained are shown in Figure 11. The abscissa indicates the driving frequency, while the ordinate indicates the output-input voltage ratio. The results show that the voltage ratio increases with frequency below 150 kHz and decreases from 150 kHz to 1 MHz. It is considered that the self-resonant frequency (SRF) of the primary coil is almost 150 kHz (the red point in Figure 11). However, the sampling frequency of the DAQ is 240 kHz. In order to acquire enough sampling points for the FFT operation, the driving frequency is set to be 4 kHz (the orange point in Figure 11) to make sure there are 60 sampling points in a time period. In another way, the eddy current effect and magnetic hysteresis of the ferromagnetic plate will introduce the non-linear factors with the increase of frequency.

**Figure 12:**
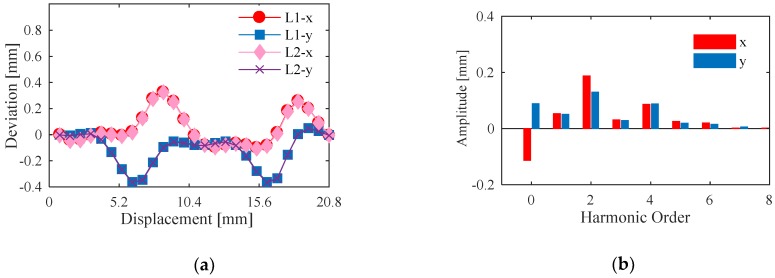
Measurement results of the sensor prototype. (**a**) Comparison of deviations in two pitches along x- and y-axis. (**b**) Harmonic distribution of deviations in one pitch.

With the motion of 2D positioning stage, the measurement values of the grating and sensor prototype are acquired synchronously. The given motion path of the stage is *y* = *x*, while the displacements in *x*- and *y*-axis are two pitches, and the linear steps are 0.8 mm, respectively. Taking the outputs of two optical gratings as the standard values, the deviations between the sensor prototype and the gratings are shown in Figure 12a. The ″L1″ in the legend indexes the one pitch, and ″L2″ indexes the other pitch. As shown as the figure, the curves of both pitches are almost the same. The maximum deviation of *x* is 0.494 mm, while the maximum deviation of *y* is 0.408 mm. The harmonic distributions of the deviations in one pitch along x- and y-axis are shown in Figure 12b. 

The main harmonic orders of the deviations are the 0th, 1st, 2nd, and 4th order. Because the initial position of the prototype is not equal to zero, the 0th harmonic components are reserved. Due to the parallelism between the primary and secondary coil is difficult to be confirmed, the lift-offs of the four spiral coils of secondary coil differ from each other. The lift-off affects not only the amplitudes of the output signals, but also the phases [31,32]. Then the amplitudes of four roads of EMFs are different. Meanwhile, the phase diversities among four roads of EMFs are not consistent. Therefore, the 2nd and 4th harmonic components are introduced. In another way, the parallel misalignment between the motion axis of the positioning stage and the sensor prototype leads to the Abbe errors during the displacement measurements of *x* and *y*. Consequently, the 1st harmonic component is brought in. Due to the legible harmonic components, error compensation can be utilized to improve the linearity.

## 5. Conclusions

This paper introduces a novel 2D inductive sensor which is based on the principle of electromagnetic induction. The sensor consists of a primary coil and a secondary coil. The primary coil is composed of a planar array of spiral coils whose successive spiral coils are wound in alternate directions. All the spiral coils are connected in series and are supplied with alternating current. Thereby, an array of pulsating magnetic field is generated. Secondary coil is composed of four spiral coils which are arranged as a 2 × 2 matrix, and the center to center distance between two successive spiral coils is 0.75 W. Hence, four roads of signals are acquired. Then, a resolving algorithm which is based on the CORDIC algorithm is proposed. The structure and working principle of the sensor are described, and verified by the FEA software and numerical simulation. A sensor prototype with 20.8 mm pitch and 133 mm × 150 mm measurement range is fabricated. The linearity in x-axis is 0.37%, while it is 0.27% in y-axis. Through all the works above, it shows that the proposed scheme for the 2D displacement measurement is reasonable and feasible.

On the other hand, there are many reasons which may lead to errors. The parallelism between primary and secondary coil affects the amplitude differences of four roads of signals. Consequently, the second and fourth order errors are introduced. In addition, the first order error will be introduced because of the parallel misalignment between the motion axis of positioning stage and the sensor prototype.

In the following work, the automatic gain control will be utilized to make the amplitudes of four roads of signals equal. Moreover, a compensation technique may be used to reduce the lift-off effect. Furthermore, a much more precise positioning stage and calibration method will be adopted to avoid the influence of Abbe error of calibration. Thus, a better linearity will be realized.

## Figures and Tables

**Table 1:** Parameters of the sensor model.

Parameter	Settings
Turns of each spiral coils of primary coil	5 turns
Turns of Sc1,Sc2,Sc3,Sc4	10 turns
Resistance of Sc1,Sc2,Sc3,Sc4	1 GΩ
Amplitude and frequency of the current in primary coil	0.1 A/4kHz
Start and stop time of simulation	10 μs, 300 μs
Material of the ferromagnetic plate	Steel_1045
Size of the ferromagnetic plate	75 mm × 75 mm × 1 mm
Material of the coils	copper
Size of the primary coil	64.6 mm × 64.6 mm
Size of the secondary coil	26 mm × 26 mm
Number of spiral coils of primary coil	6 × 6
Conductor width\space\thickness	0.1 mm
Center distance of two successive spiral coil of primary coil	10.4 mm
Length\width of the outmost turn of spiral coil of primary coil	5 mm
Length\width of the outmost turn of spiral coil in Sc1~Sc4	5.2 mm
Center distance between Sc1 and Sc2	15.6 mm
Center distance between Sc1 and Sc4	15.6 mm
Air-gap thickness between the spiral coils and the ferromagnetic plate	0.1 mm
Air-gap thickness between the primary and secondary coil	0.1 mm
Moving path of the secondary coil	*y* = *x*
Displacements and linear step along x- and y-axis	20.8 mm, 0.8 mm

**Table 2:** Interval judgment.

No.	Condition	Condition	*α* and *β*
Ⅰ	AA>BB&&BB>=0 &&|AA|>|BB	a(0)=|BB|; b(0)=|AA|	*α*, *β*=c(50)
Ⅱ	BB>=AA&&AA>=0 &&|BB|>|AA|	a(0)=|AA|;b(0)=|BB|	*α*, *β*=π/2−c(50)
Ⅲ	BB>=0&&AA<0 &&|BB|>=|AA|	a(0)=|AA|;b(0)=|BB|	*α, β=π/2*+c(50)
Ⅳ	BB>=0&&AA<0 &&|AA|>|BB|	a(0)=|BB|;b(0)=|AA|	*α, β*=π−c(50)
Ⅴ	BB<0&&AA<BB &&|AA|>=|BB|	a(0)=|BB|;b(0)=|AA|	*α, β*=π+c(50)
Ⅵ	AA<0&&AA>=BB &&|BB|>=|AA|	a(0)=|AA|;b(0)=|BB|	*α, β*=3π/2−c(50)
Ⅶ	AA>=0&&BB<0 &&|BB|>=|AA|	a(0)=|AA|; b(0)=|BB|	*α, β*=3π/2+c(50)
Ⅷ	AA>=0&&BB<0 &&|AA|>|BB|	a(0)=|BB|; b(0)=|AA|	*α, β*=2π−c(50)

**Table 3:** Cross-border judgments.

No.	Condition	*n_α_* and *n_β_*
Ⅰ	(ab1−ab0)<−(2π−π/20) && (ab1−ab0)> −2π && (ba1−ba0)< − (2π−π/20) && (ba1−ba0)> −2π	*n_α_*= *n_α_*+1; *n_β_*= *n_β_*+1
Ⅱ	(ab1−ab0)<2π && (ab1−ab0)>(2π−π/20) && (ba1−ba0)<2π && (ba1−ba0)>(2π−π/20)	*n_α_*= *n_α_*−1;*n_β_*= *n_β_*−1
Ⅲ	(ab1−ab0)<2π && (ab1−ab0)>(2π−π/20) && (ba1−ba0)< − (2π−π/20) && (ba1−ba0)>2π	*n_α_*= *n_α_*−1;*n_β_*= *n_β_*+1
Ⅳ	(ab1−ab0)< − (2π−π/20) && (ab1−ab0)> −2π && (ba1−ba0)<2π && (ba1−ba0)>(2π−π/20)	*n_α_*= *n_α_*+1; *n_β_*= *n_β_*−1
Ⅴ	(ab1−ab0)<2π && (ab1−ab0)>(2π−π/20) && |ba1−ba0|<π/20	*n_α_*= *n_α_*−1; *n_β_*= *n_β_*
Ⅵ	|ab1−ab0|<π/20 && (ba1−ba0)< π/20 && (ba1−ba0)>(2π−π/20)	*n_α_*= *n_α_*; *n_β_*= *n_β_*−1
Ⅶ	(ab1−ab0)< − (2π−π/20) && (ab1−ab0)> −2π && |ba1−ba0|<π/20	*n_α_*= *n_α_*+1; *n_β_*= *n_β_*
Ⅷ	|ab1−ab0|<π/20 && (ba1−ba0)< − (2π−π/20) && (ba1−ba0)> −2π	*n_α_*= *n_α_*; *n_β_*= *n_β_*+1
